# 1575. Comparison of virologic suppression rates pre-, intra- and postpartum in women with HIV in an underserved US community

**DOI:** 10.1093/ofid/ofad500.1410

**Published:** 2023-11-27

**Authors:** Elise N Gerdes, Jin S Suh, Chloe Kim, Humberto R Jimenez

**Affiliations:** Ernest Mario School of Pharmacy, Princeton, New Jersey; St. Joseph's University Medical Center, Ridgewood, New Jersey; Ernest Mario School of Pharmacy, Princeton, New Jersey; Rutgers University / St. Joseph's University Medical Center, Paterson, New Jersey

## Abstract

**Background:**

Antiretroviral therapy (ART) has significantly reduced mother-to-child transmission of HIV among pregnant women. Nonetheless, numerous factors can impede ART adherence and thus virologic suppression (HIV RNA < 200 copies/mL). Although there are limited data on current rates of virologic control in women with HIV around pregnancy, previous studies have highlighted lower rates at the start of pregnancy and 6 months postpartum. The objective of this study was to compare virologic suppression rates in women pre-pregnancy, intrapartum, and postpartum at a clinic serving a marginalized community and to identify factors associated with virologic control

**Methods:**

This study was a single-center, retrospective chart review including pregnant patients receiving care at a hospital-based, NJ Ryan-White funded clinic between 2015-2022. The primary endpoint was to determine whether there is a difference in the proportion of patients with virologic suppression at the start of pregnancy, at delivery, and 6 months postpartum

**Results:**

At the first visit, 64.4% of mothers were virologically suppressed. Our study found that the proportion of women remaining virologically suppressed was lower at 6 months postpartum compared to the time of delivery (82.2% versus 90.4%, p=0.017), regardless of virologic suppression at the first visit. Older age at pregnancy was associated with improved virologic suppression at 6 months postpartum (OR 1.13, 95% CI 1.01-1.27). However, perinatal HIV acquisition, as compared to heterosexual acquisition, was correlated with increased virologic failure at 6 months postpartum (OR 5.4, 95% CI 1.42-20.08).

**Figure:**
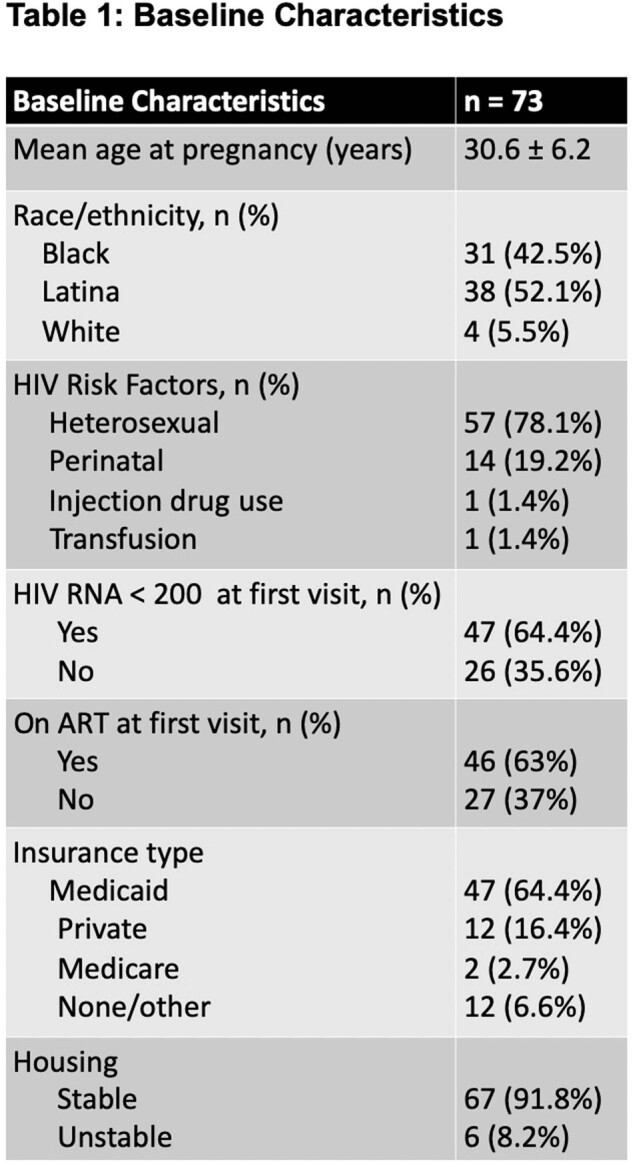

**Figure:**
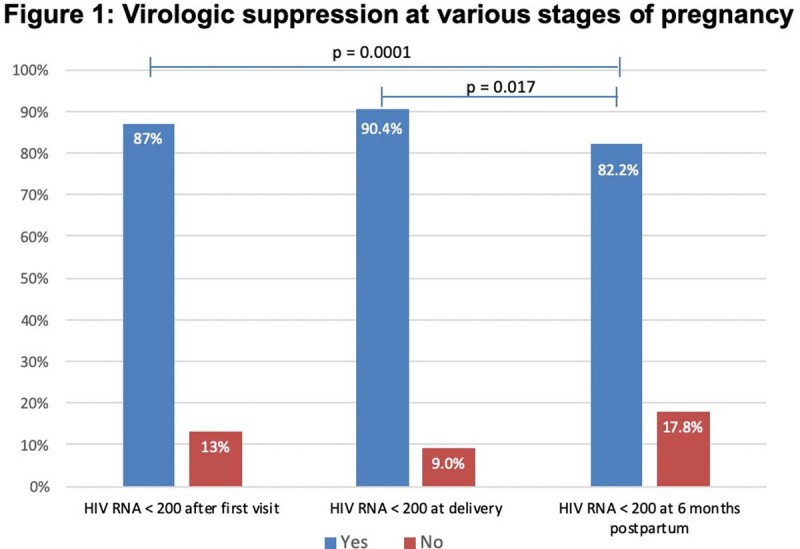

**Conclusion:**

Although a high percentage of patients were controlled at delivery, the drop in virologic suppression postpartum highlights the need for additional efforts to support the mother and child during that time. While further research is warranted to identify further confounders that may impact virologic control and other barriers to care, the healthcare team should work together holistically on frequent outreach and follow up during the entire pregnancy.

**Disclosures:**

**All Authors**: No reported disclosures

